# How to select cancer patients for immunotherapy

**DOI:** 10.1016/j.ebiom.2020.103184

**Published:** 2021-01-06

**Authors:** Per Pfeiffer, Camilla Qvortrup, Karin Holmskov Hansen

**Affiliations:** a Department of Oncology, Odense University Hospital, Sdr Boulevard 29, Odense C 5000, Denmark; b Department of Oncology, Rigshospitalet, Copenhagen University, Blegdamsvej 9, Copenhagen 2100, Denmark

In recent years, the importance and understanding of immunotherapy in the treatment of solid tumours has increased dramatically. Presently, immune checkpoint inhibitors (ICIs) are the most widely used immunotherapy and are approved for several indications across different solid malignancies, such as malignant melanoma, non-small cell lung carcinoma, renal cell carcinoma, and bladder cancer [1]. In addition, ICI have shown considerable activity in microsatellite instable tumours (MSI), irrespective of the tissue of origin [2,3]. In 2017, the US FDA granted approval of pembrolizumab for chemorefractory patients with MSI solid tumours, but this tumour-agnostic indication has not yet been submitted for approval in Europe.

However, despite the higher response rate and longer progression-free survival, it is still only a minority of patients that will experience a long-lasting response and therefore exact and validated predictive biomarkers are necessary. The programmed death 1 (PD-1) receptor is a cell surface inhibitory receptor expressed on immune cells like T cells and NK cells. PD-1 has two known ligands - PD-L1 and PD-L2. PD-L1 is often upregulated in malignant cells and in the surrounding tumour microenvironment. PD-L1 inhibits and downregulates T cell response by interacting with the PD-1 receptor but inhibition of PD-1 or PD-L1 by ICI may restore T-cell activity. Logically, PD-L1 expression correlates with clinical outcomes and ever since the earliest trials showed an association with efficacy, the PD-L1 expression has been an unavoidable part of many ICI trials [1,3]. Unfortunately, there is no universal consensus on the definition of a PD-L1 positive tumour, and several scorings systems like TPS (tumor Proportion Score) and CPS (Combined Positive Score) are in clinical use. In addition, a recent meta-analysis concluded that presently PD-L1 expression status alone is insufficient for the decision of which patients should be offered ICI [4].

The molecular changes with direct implication on the choice of therapy are PD-L1 and MSI status, but in clinical practice, it is often debated whether molecular analysis should be performed on tissue from the primary or from metastatic tissue.

Therefore, the present comprehensive meta-analysis published in this issue of *EBioMedicine* is of great importance [2]. The authors evaluated predictive biomarkers for immunotherapy benefit, focusing on the concordance of the expression of these markers between the primary lesions and paired metastases. Data from 2739 patients in 56 studies of which the most frequent primaries were lung (*n* = 715), breast (*n* = 366), and kidney cancer (*n* = 325) showed an excellent agreement for MSI-status. In contrast, they demonstrated a substantial discordance when evaluating PD-L1 (pooled discordance rate 22%), PD-L2 (22%), PD-1 (26%) and TIL (39%) with a huge variety in discordance rate. Specifically focusing on the concordance of PD-L1 in 38 studies with 2109 patients, the percentage that changed from positive to negative was 41%, and from negative to positive was 16%.Therefore, the recommendation from the authors was to evaluate these biomarkers in both the primary and metastases.

Many commercial antibodies are available for measuring PD-L1, and four different antibodies have been in use for development of different ICIs; pembrolizumab (antibody 22C3), nivolumab (28–8), atezolizumab (SP142), and durvalumab (SP263). In the present meta-analysis as many as 10 different antibodies were used to evaluate PD-L1 expression and in addition 9 different cut-off points were used, all of which make interpretation complex. To harmonize results, recent studies have shown comparable results for some of the most widely used antibodies (22C3, 28–8, and SP263) [3]. Hopefully, it will be possible to use these antibodies and assays interchangeably in the future.

In another recent meta-analysis evaluating PD-L1 expression [5], the authors included only 13 trials (451 patients, all retrospective trials) and it is remarkable that only 8 of these trials were also included in the present meta-analysis [2]. The pooled discordant rate in PD-L1 expression was 31% with high heterogeneity across the studies. Lee et al. found no significant correlation between PD-L1 discordant rates and PD-L1 status of the primary (positive 42%, negative 22%), PD-L1 positivity threshold, origin of the primary, site of metastatic disease, timing of metastasis (synchronous 44%, metachronous 22%), or types of antibody clones used.

At the current time, MMR/MS-status can be evaluated both on the primary and metastases and the clinical decision can be made based on these results. However, for assessment of PD-L1, presently there is no standardized assay. Furthermore several different antibodies, cut-off points and scoring systems have been applied not only across the different tumour types but also within the same primary and the specific ICI. It is still not known if PD-L1 is best evaluated on the primary or metastatic lesion. This lack of harmonization compromises the interpretation of the results of the different trials, but importantly it also compromises the daily clinical utility. In conclusion, the interpretation and use of PD-L1 expression is complicated and there are many unsolved questions - including the understanding of the heterogeneity on PD-L1 expression and clinical.

## Author contribution

The first draft was written by Per Pfeiffer and then read, discussed, and corrected by all authors. All authors approved the final version of the commentary.

## Declaration of Competing Interest

Dr. Qvortrup reports grants from Servier, grants from Pierre-Fabre, personal fees from PledPharma, personal fees from Roche, personal fees from Merck, other from Incyte, other from Bayer, outside the submitted work; Dr. Hansen and Prof. Pfeiffer have nothing to disclose.

## References

[1] Zhao B., Zhao H., Zhao J. (2020). Efficacy of PD-1/PD-L1 blockade monotherapy in clinical trials. Ther Adv Med Oncol.

[2] Zou Y., Hu X., Zheng S. (2020). Discordance of immunotherapy response predictive biomarkers between primary lesions and paired metastases in tumours. EBioMedicine.

[3] Duffy M.J., Crown J. (2019). Biomarkers for predicting response to immunotherapy with immune checkpoint inhibitors in cancer patients. Clin Chem.

[4] Shen X., Zhao B. (2018). Efficacy of PD-1 or PD-L1 inhibitors and PD-L1 expression status in cancer: meta-analysis. BMJ.

[5] Lee C.C., Soon Y.Y., Lum J.H.Y., Tan C.L., Tey J.C.S. (2020). Frequency of discordance in programmed death-ligand 1 (PD-L1) expression between primary tumors and paired distant metastases in advanced cancers: a systematic review and meta-analysis. Acta Oncol.

